# Motor development-focused exercise training enhances gross motor skills more effectively than ordinary physical activity in healthy preschool children: an updated meta-analysis

**DOI:** 10.3389/fpubh.2024.1414152

**Published:** 2024-05-21

**Authors:** Xinchen Wang, Bo Zhou

**Affiliations:** College of Physical Education, Hunan Normal University, Changsha, China

**Keywords:** early childhood, fundamental movement skills, growth, pediatrics, physical education

## Abstract

**Purpose:**

The growth of certain human brain structures peaks at early ages, and complex motor interventions could positively facilitate this process. This study aims to offer an updated meta-analysis regarding the effectiveness of motor development-focused exercise training on gross motor skills in preschool children.

**Methods:**

We searched English- and Chinese-language electronic databases as of March 2024. The main eligibility criteria were as follows: participants were healthy children aged 3 to 6 years old, and the experimental design was a randomized controlled trial, with the control arm participating in either free play or ordinary physical education curriculum. We conducted a Hartung-Knapp random-effects meta-analysis of the standardized mean difference for locomotor, object control, or gross motor quotient.

**Results:**

The search identified 23 eligible studies, of which approximately 75% were considered to have a low risk of bias. Compared with active control, exercise training showed a large to very large effect size. Cohen’s d values were 1.13, 1.55, and 1.53 for locomotor, object control, and gross motor quotient, respectively. From a probabilistic viewpoint, these effect sizes correspond to events that are “very likely to occur” and “almost sure to occur.” Due to variations in intervention programs, all outcome measures showed high heterogeneity.

**Conclusion:**

This updated meta-analysis offers a realistic synthesis of the current evidence, leading to the conclusion that targeted motor skill exercise training can almost certainly enhance preschool children’s gross motor skills. Practical implications are discussed regarding the refinement of the instructional framework and the dissemination of these findings in preschool settings.

## 1 Introduction

Not only are motor skills important for children’s physical development, but a wealth of literature also demonstrates that they serve as crucial developmental catalysts for executive functions, prosocial behaviors, and intellectual abilities (1, 2). Failure to develop motor proficiency in early childhood can have lifelong consequences. Evidence suggests that motor development promotes neurocognition, occurring in brain regions such as the cerebellum and prefrontal cortex (3). Meanwhile, human brain development peaks in early life; for instance, Lobule IX and Crus II of the cerebellum mature at 5 and 7 years old (4), respectively, and are correlated with motor and cognitive functions (5). Therefore, early childhood complex motor intervention programs could lead to improved brain morphology in childhood (6) and enhanced prospects later in life (7). In this context, preschool children, typically aged 3 to 6 years old, form a unique intervention cohort as they are often in daycare settings that lack formalized physical activity guidelines, leading to difficulties in meeting recommended physical activity levels (8).

It is useful to first distinguish between two types of motor skills. Fine motor skills involve the coordination of the hands, fingers, and wrists for tasks like grabbing, holding, and manipulating objects, as well as visual motor coordination. On the other hand, gross motor skills, often referred to as fundamental movement skills, encompass basic movements such as locomotor (e.g., hopping) and object control (e.g., ball handling). While both fine and gross motor skills are crucial for children’s all-round development, current evidence indicates that gross motor skills demonstrate greater predictive power regarding children’s high-functioning brain structures (9) and neurocognitive development (10). Accordingly, physical education (PE) programs that focus on developing children’s gross motor skills are of great value in preschool settings.

While randomized controlled studies on gross motor interventions in children have been accumulating slowly over the past decades, current studies suggest that motor development-focused exercise training is effective in improving children’s gross motor skills. Nevertheless, there are two critical shortcomings in the existing literature. Firstly, several studies and literature reviews compare intervention programs to passive control (i.e., no physical activity) (11–13), which does not accurately reflect realistic settings. For example, in China, the Ministry of Education suggests that all preschools ensure children have 2 hours of outdoor activities per day, with a minimum of 1 hour dedicated to physical activities. While it may be true that daily physical activity cannot be guaranteed in some kindergartens or daycare centers due to environmental or socioeconomic factors, most modern schools allocate physical activities for children’s optimal physical development. Consequently, studies designed with a passive control group offer limited insight into the efficacy of motor intervention programs. Secondly, although a recent systematic review (12) conducted a meta-analysis of intervention programs on children’s gross motor skills and extended findings from two earlier meta-analyses (13, 14), it did not incorporate several new evidence, compromising an already small pool of literature in this field. Zhang et al. included only seven studies that measured gross motor skills and concluded that motor intervention programs have a large effect size (Cohen’s d = 0.889) in preschool children. However, it missed several high-quality studies that reported very large effect sizes of motor intervention programs (15) (see also the complete citation list in Results). As a result, conclusions from the existing meta-analysis (12–14) did not fully demonstrate the value of motor intervention programs.

A pressing question arises: Is targeted exercise training more advantageous than free play or an ordinary PE curriculum in promoting gross motor skills in preschool children? This question is substantiated by two key justifications. Firstly, preschool children are undergoing a period of rapid growth in both the structure and functionality of their brains, as aforementioned. Therefore, it is essential to implement targeted intervention programs for preschool children. Secondly, it is imperative to establish an efficient instructional framework. While routine daycare in kindergartens does provide time for children to engage in physical activities, these activities are often unstructured or follow an ordinary PE curriculum, such as radio gymnastics in China. Motor proficiency is an outward manifestation of one’s executive functions. Current research indicates that young children show higher levels of motivation to enhance their cognitive abilities when guided by a teacher or involved in a well-structured program (16). Thus, the active education model proposes that teacher-directed activities may be more effective in developing executive functions compared to liberal programs (17). Hence, it is crucial to understand the efficacy of these two types of instructional frameworks in a typical preschool setting. While most data supports the effectiveness of targeted exercise training, there is also negative evidence suggesting that ordinary physical activity is equally effective in enhancing preschool children’s gross motor skills (18). Analyzing the collective effect of targeted exercise training through meta-analysis can contribute to creating a scientific PE curriculum aimed at optimizing young children’s brain development.

Therefore, the purpose of this study is to provide an updated meta-analysis of the efficacy of motor development-focused exercise training on gross motor skills compared to active control in typical preschool settings. To expand the available evidence, this meta-analysis also includes high-quality research published in the Chinese language. Ultimately, the goal is to provide up-to-date evidence to guide an effective PE curriculum for preschool children.

## 2 Methods

### 2.1 Search strategy

We conducted an electronic database search across multiple databases, including MEDLINE, Web of Science’s Core Collection, the Cochrane Central Register of Controlled Trials, and Wanfang Data, from their inception to March 2024. Our search utilized English and Chinese keyword combinations as follows: (“fundamental motor skill*” OR “fundamental movement skill*” OR “gross motor”) AND (“physical activity”, “exercise”, “training”); (“动作技能” [“fundamental motor skill”] OR “粗大动作” [“gross motor”]) AND (“运动” [“exercise”], OR “训练” [“training”]). Additionally, we reviewed citations from four systematic reviews and included relevant studies.

### 2.2 Eligibility criteria

The inclusion criteria were as follows: participants were healthy children aged 3 to 6 years old; the intervention was an exercise training program aimed at enhancing preschool children’s gross motor skills; the outcomes included at least one gross motor skill metric; and the experimental design was a randomized controlled trial. The exclusion criteria were as follows: studies that enrolled children presenting physical disability, intellectual or developmental disorders; reviews, conference abstracts, protocols, or cross-sectional studies; and the control arm involved with no confirmed physical activity. Two authors independently screened the records, resolving disagreements by consensus.

### 2.3 Outcomes

The outcomes of interest in this meta-analysis were gross motor skills. Commonly used assessments for these skills include the Test of Gross Motor Development 2nd/3rd Edition (TGMD-2/3) or the Peabody Developmental Motor Scales 2nd Edition (PDMS-2). The TGMD-2/3 comprises two tests: locomotor, which evaluates children’s gross motor skills involving smooth and coordinated movements in one direction, and object control (referred to as ball skills in the 3rd Edition), which assesses children’s ability to throw, strike, and catch objects efficiently. The gross motor quotient is derived from summing the scores of these two tests. In this meta-analysis, all three metrics of the TGMD-2/3 and the gross motor score of the PDMS-2 were considered as outcome measures.

### 2.4 Data extraction

The first author extracted data using a standardized spreadsheet. The extracted dataset can be assessed on Figshare (DOI: 10.6084/m9.figshare.25545703.v1). The certainty of evidence was evaluated using the RoB 2 tool (19), which is organized into five bias domains: bias arising from the randomization process, bias due to deviations from intended interventions, bias due to missing outcome data, bias in the measurement of the outcome, and bias in the selection of the reported result. Two authors independently assessed potential biases for each study and resolved any discrepancies through discussion.

### 2.5 Data synthesis and analysis

This meta-analysis was conducted using the R package meta version 7.0-0. Among the included studies, one study reported findings separately for males and females (20), while another study provided data for different age groups (4 and 5 years old) (21). To reduce the potential impact of bias from a single study on the overall effect, data from two separate groups within each study were combined to generate a composite score. This composite score was calculated using Eq. 1:


(1) $$\begin{array}{l} \mathrm{Ncombined}=N1+N2\\ \mathrm{Meancombined}=\frac{N1M1+N2M2}{N1+N2}\\ \mathrm{SDcombined}=\sqrt{\frac{\begin{array}{l} \left(N1-1\right)SD1^{2}+\left(N2-1\right)SD2^{2}+\\ \frac{N1N2}{N1+N2}\left(M1^{2}+M2^{2}-2M1M2\right) \end{array}}{N1+N2-1}} \end{array}$$

We pooled data as standardized mean difference (Cohen’s d) and 95% confidence interval. Due to the diverse nature of exercise training in terms of intensities, duration, and instructional methods, the between-study variance cannot be solely assumed from sample variation. Hence, we employed the Hartung-Knapp random-effects model. The I^2^ statistic was utilized to gauge the proportion of total variability attributed to heterogeneity between the studies. Furthermore, publication bias was evaluated using Egger’s regression test along with a visual examination of the funnel plot. To facilitate the dissemination of our research to the general public, we further converted the effect size to a common-language effect size using Eq. 2 (22):


(2) $$\mathrm{Common}\;\mathrm{language}\;\mathrm{effect}\;\mathrm{size}=\Phi \left(\frac{d}{\sqrt{2}}\right)$$

where, Ф is the normal cumulative distribution function.

## 3 Results

### 3.1 Search results

**Figure 1:**
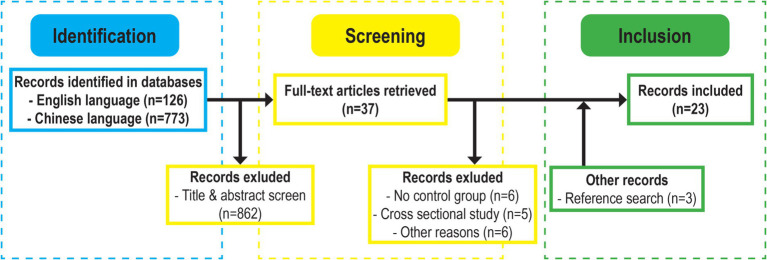
Flow chart of the literature search.

Figure 1 illustrates the search process. As of March 2024, our search strategy yielded a total of 899 records after removing duplicates. Two researchers independently assessed 37 full-text articles, resulting in the removal of 11 records due to the absence of control groups or the cross-sectional nature of the study. Another six records were excluded due to methodological issues. While Foulkes et al.’s study (23) met all eligibility criteria, their statistical reporting cannot be used for the calculation of effect size. Calero-Morales et al. examined the effects of a conductive educational model compared to a constructive model on fundamental motor development (24) but lacked crucial details, such as information on PE curricula, affecting the research’s quality and result validity. Melvin Chung et al.’s study (25) lacked clarity regarding the control group’s inclusion of a physical education class and had incomplete statistical reporting (i.e., the lack of reporting standard deviations) for post-analysis. In Rocha et al.’s study (26), it was unclear if the control group received ordinary physical activity in kindergarten. Lee et al. (27) reported a subset of data from their publication (28), which was included in this meta-analysis. Mostafavi et al.’s study (29) lacked explicit information on sample sizes for both intervention and control groups, making it unsuitable for data synthesis.

### 3.2 Included studies

This meta-analysis included a total of sixteen studies published in English literature (15, 18, 20, 21, 28, 30–39) and seven studies published in Chinese literature (40–46). Table 1 summarizes the key characteristics of the included studies. In total, this meta-analysis included 1,074 children enrolled in the exercise training group and 996 children enrolled in the control group. Except for one study that only enrolled female participants (18), the remaining studies included both male and female participants. Among the sixteen studies that reported the gender distribution, the proportions of males and females were found to be 49.1 and 50.9%, respectively. Nineteen studies evaluated gross motor skills using the TGMD-2, while four studies used the TGMD-3, and one study used the PDMS-2 (34).

**Table 1:** Characteristics of exercise training from included studies.

Source	Exercise volume	Exercise activity
Robinson and Goodway (30)	30-min/sess × 2 sess/wk. × 9 wks	Semi-instructed tasks [FP]
Jones et al. (31)	20-min/sess × 3 sess/wk. × 20 wks	Teacher-led “Jump Start” [FP]
Bardid et al. (32)	60-min/sess × 2 sess/wk. × 12 wk	Teacher-led training [PE]
Jones et al. (33)	20-min/sess × 3 sess/wk. × 6 mon	Teacher-led “Jump Start” [FP]
Zhou et al. (40)	40–50-min/sess × 5 sess/wk. × 12 wks	Teacher-led functional training [PE]
Hamilton and Liu (34)	25-min/sess × 2 sess/wk. × 16 wks	1-teacher-4-children training [PE]
Lei et al. (41)	60-min/sess × 3 sess/wk. × 8 wks	Teacher-led functional training [PE]
Roach and Keats (35)	45-min/sess × 2 sess/wk. × 8 wks	“Successful Kinesthetic Instruction” [FP]
Zha et al. (42)	60-min/sess × 2 sess/wk. × 12 wks	Gymnastics [PE]
Bolger et al. (36)	25–30-min/sess × 2 sess/wk. × 26 wks	Teacher-led movement skills [PE]
Johnson et al. (47)	45-min/sess × 29 sess over 9 mon	Semi-instructed play [FP]
Palmer et al. (37)	40-min/sess × 3 sess/wk. × 5 wks	Teacher-led “CHAMP” [FP]
Hu et al. (21)	One academic year	Rhythmic activities and games [PE]
Lee et al. (28)	60-min/sess × 3 sess/wk. × 8 wks	Teacher-led training [FP]
Marinšek and Denac (38)	40-min/sess × 4 sess/wk. × 5 wks	Music and movement program [PE]
Gu et al. (20)	50-min/sess × 3 sess/wk. × 12 wks	Theme-plot table tennis training [PE]
Wen et al. (43)	30–40-min/sess × 3 sess/wk. × 8 wks	Teacher-led, theme-plot training [PE]
Engel et al. (39)	40-min/sess × 1–5 sess/wk. × 12 wks	“PLAYFun” program [FP]
Fu et al. (15)	40–50-min/sess × 1–2 sess/wk. × 12 wks	Teacher-led functional training [FP]
Gu and Li (44)	50-min/sess × 3 sess/wk. × 12 wks	Games and table tennis practice [PE]
Abusleme-Allimant et al. (18)	45-min/sess × 1 sess/wk. × 12 wks	Structured tasks [FP]
Liu and Fan (45)	12 wks	Figure rope skipping [PE]
Liu et al. (46)	90-min/sess × 1 sess/wk. × 12 wks	Happy gymnastics [PE]

Physical activity of control group in []. sess, session; wk, week; mon, month; FP, free play; PE, physical education.

**Figure 2:**
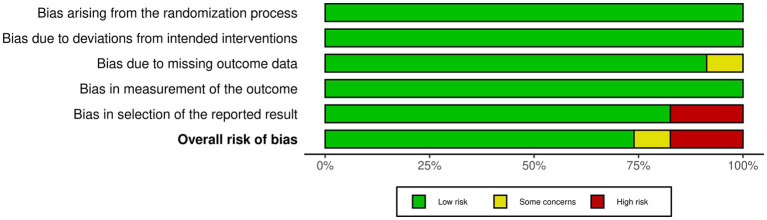
Risk of bias in included studies.

Figure 2 illustrates the assessment of bias risk. Overall, approximately 75% of the included studies were classified as having a low risk of bias. However, despite Hamilton and Liu (34), as well as Engel et al. (39), utilizing a randomized controlled research design, these studies did not include baseline data, raising concerns about outcome measurement (i.e., RoB 2 “3.2 Is there evidence that the result was not biased by missing outcome data?”). Robinson and Goodway only assessed object control, introducing bias in the selection of the reported result (i.e., RoB 2 “5.2 Is the numerical result being assessed likely to have been selected, on the basis of the results, from multiple eligible outcome measurements (eg, scales, definitions, time points) within the outcome domain?”). Similarly, Jones et al. (31, 33) and Marinšek and Denac (38) reported only a subset of locomotor and object control, leading to a high risk of bias in the selection of the reported result (i.e., RoB 2 “5.3 Is the numerical result being assessed likely to have been selected, on the basis of the results, from multiple eligible analyses of the data?”).

### 3.3 Effect of exercise training

**Figure 3:**
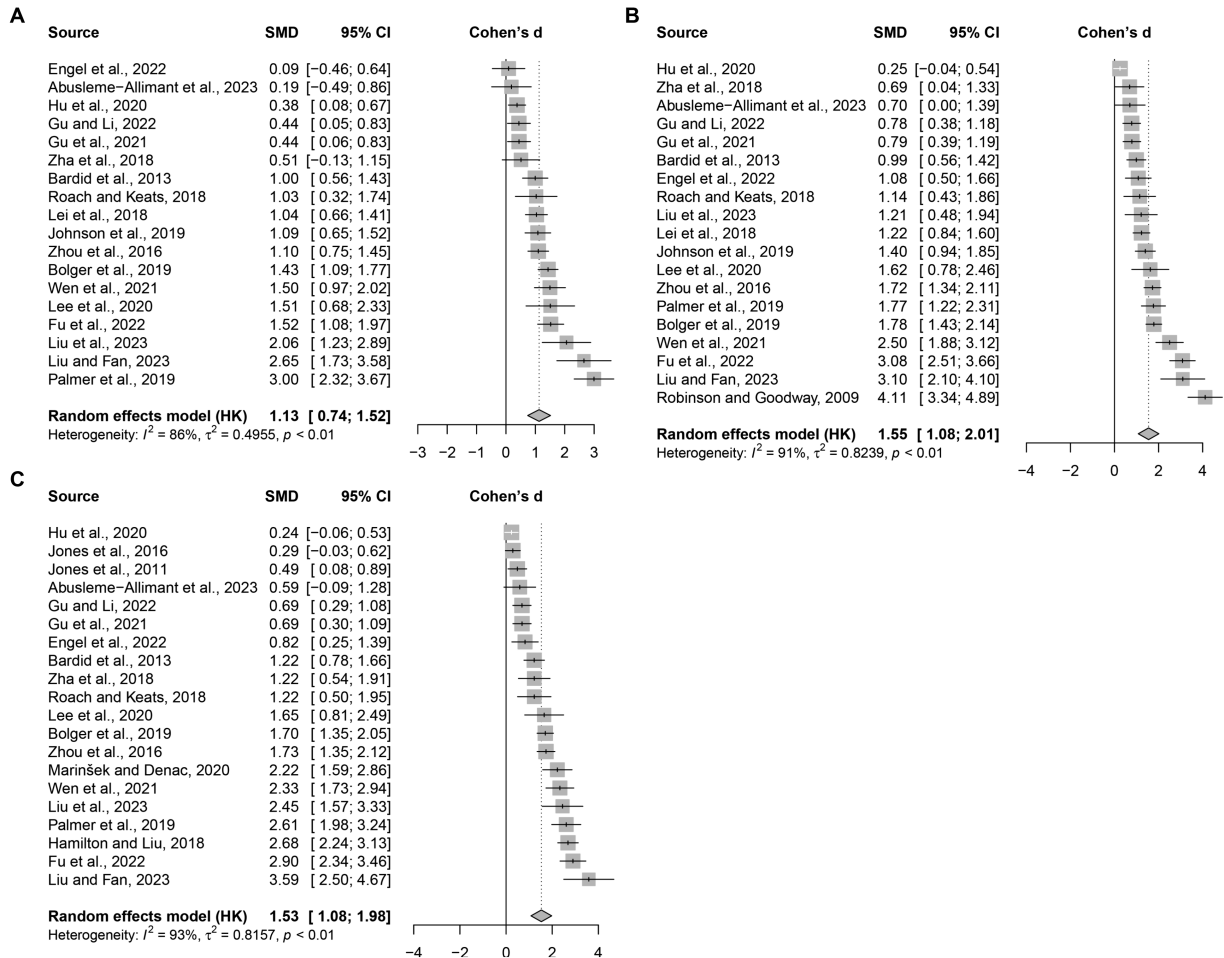
Cohen’s d values of studies comparing gross motor skill development between exercise training and ordinary physical activity among 3–6 years old children: **(A)** locomotor; **(B)** object control; **(C)** gross motor quotient. Each square represents the observed effect size of a study, with its size corresponding to the study’s weight. The diamond on the plot represents the pooled effect size.

Figure 3 illustrates the pooled effect size. Locomotor scores were reported in eighteen studies, revealing a large effect size for exercise training compared to free play or PE curriculum. Object control scores were reported in nineteen studies, indicating a very large effect size for exercise training compared to both free play and PE curriculum. Overall, exercise training demonstrates superiority (Cohen’s d = 1.53, 95% CI 1.08 to 1.98) over free play or PE curriculum in developing gross motor skills in preschool children. Based on the common-language effect size, exercise training has a 78.8, 86.3, and 86.0% probability of being superior to free play or PE curriculum in enhancing locomotor, object control, and gross motor quotient, respectively. It is noteworthy that all three outcomes show a high I^2^ statistic, indicating significant variation among the included studies.

**Figure 4:**
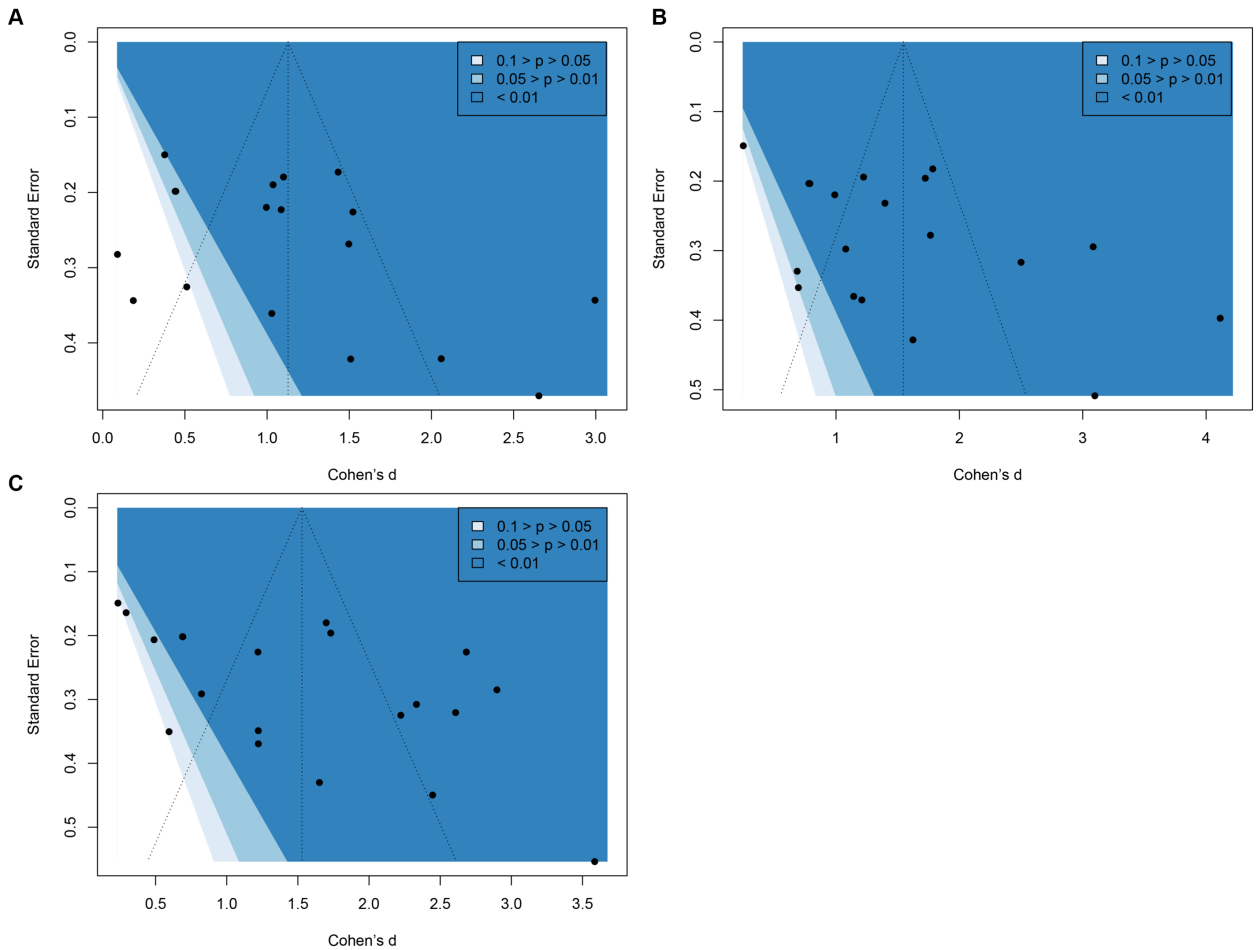
Contour-enhanced funnel plot: **(A)** locomotor; **(B)** object control; **(C)** gross motor quotient. Each circle on the plot represents an included study. The vertical line indicates the overall Cohen’s d.

Based on Egger’s regression test, the *p*-values for locomotor, object control, and gross motor quotient were 0.09, 0.02, and 0.01, respectively. This indicates statistically significant publication bias in object control and gross motor quotient. However, it raises the question of whether this bias reflects non-significant studies not being published or a genuinely superior effect of exercise training. A visual examination of the funnel plot (Figure 4) suggests that most studies exhibit highly statistically significant effects (*p* < 0.01) of exercise training compared to free play or PE curriculum. Given this substantial evidence favoring motor development-focused exercise training, we conclude that there is no publication bias among the included studies.

## 4 Discussion

Our meta-analysis revealed that compared to free play or PE curriculum, exercise training demonstrates a large to very large effect size in developing gross motor skills in preschool children. It is important to begin by discussing how our meta-analysis updates previous findings (12–14) and the main practical implication of these results. Van Capelle et al. (14), based on nine studies, reported an effect size (Cohen’s d) of intervention programs on gross motor proficiency over passive or active control of 0.13, corresponding to a common-language effect size of 53.7%. Wick et al. (13), based on thirteen studies, found an effect size (Cohen’s d) of 0.46, corresponding to a common-language effect size of 62.8%. Zhang et al. (12), based on seven studies, reported an effect size (Cohen’s d) of 0.889, corresponding to a common-language effect size of 73.5%. In comparison, our updated meta-analysis, based on twenty studies comparing intervention programs to active control, shows a common-language effect size of 86.0%. From a probabilistic point of view (48), 53.7, 62.8, 73.5, and 86.0% represent events of “an even chance to occur”, “somewhat greater than an even chance”, “likely to occur”, and “almost sure to occur”, respectively. Not only does this updated meta-analysis provide the most realistic synthesis of the evidence to date, but in practical terms, we can now conclude that motor development-focused exercise training offers 3- to 6-year-old children an almost certain effect to enhance gross motor skills at this critical growth stage.

Three possible mechanisms contribute to the better outcome from motor development-focused exercise training. First, the interventions were well-targeted, incorporating activities that balanced motor skill development with children’s interests. Unlike self-directed activities or general PE curriculum, these interventions were designed to seamlessly integrate gross motor movements into a diverse range of exercises and/or fun games. This approach effectively stimulates the development of motor and sensory coordination in children’s cerebral cortex during complex activities (49), resulting in significant improvements in gross motor skills. For example, Fu et al. introduced the concept of functional rehabilitation training and integrated fun games, leading to a huge effect size of 2.9 (15). It is noteworthy that Fu et al. provided an explicit description of their training program, making it highly suitable for adoption by preschools in developing countries.

Second, the active role of teachers, who serve both promotion and prevention purposes, may also be an important factor. Previous research has shown that the development of executive functions in preschool children is more effective in a mentoring situation (50). In the included studies, even though some experiments provided the same equipment, children in the active control group did not show significant motor development in the non-instructed condition (34, 47), highlighting the critical role of teacher instruction in motor skill development for preschool children. Notably, the study by Hamilton and Liu (34), used one teacher instructing four children to improve gross motor skills, showing an effect size of 2.68, which can be categorized as an “extremely sure to occur” event from a probabilistic point of view. Their instructional framework may also suggest that a small-group intervention model could be especially effective for young children experiencing delayed motor skill development.

Third, the overall amount of physical activity in the exercise training group might be greater than in free play or PE curriculum, which may also be a positive factor. For example, Lee et al. showed that daily moderate to vigorous physical activity increased moderately (Cohen’s d = 0.65; common-language effect size = 67.7%; a “likely to occur” event) under exercise training compared to free play (28). Therefore, a program aimed at enhancing gross motor skills could additionally boost the overall physical activity levels of preschool children during non-intervention periods, establishing a beneficial cycle of enhanced motor proficiency, heightened activity levels, and further improvements in motor skills.

It is worth noting that in this updated meta-analysis, as well as in previous meta-analyses, all results indicate that motor development-focused exercise training can yield larger effects for object control than locomotor skills. Children’s participation in free play or PE curriculum involves a variety of running and jumping activities, thus locomotor development can occur to some extent. However, in general, structured ball-skill activities are less often involved in free time or PE curriculum. As a result, children’s object control skills may not be effectively improved, which, in turn, affects the balanced development of gross motor skills.

Considering that the intervention programs varied in frequencies, duration, exercise intensities, and program difficulty, we employed a random-effects model to quantify the effect size. Due to these differences, this updated meta-analysis also cannot isolate the contributing factors that led to the high heterogeneity among the studies. Nevertheless, it is recommended that researchers and practitioners refer to training programs from studies that yielded an effect size greater than 2 (see also Figure 3), which indicates an “extremely sure to occur” event. While these specific programs varied in frequency and modes, the authors provided detailed instructional modalities that can be replicated in practical and other research settings. In addition, we recommend that future studies focus on two nuances to optimize interventions. Firstly, while most included studies used a frequency of two to three sessions per week, we believe that preschool children would benefit more from daily participation in targeted physical activity. Increasing the frequency of exercise training is expected to be more effective in enhancing gross motor skills. Secondly, building on the second mechanism we proposed earlier, there is a need to explore the co-teaching model (51). Early childhood education often requires frequent instruction in proper motor skills during complex physical activities. The traditional one-teacher model may struggle to cater to the individual development needs of 20–30 preschool children simultaneously. Therefore, a co-teaching model could be more conducive to the overall coordination of physical activities and the development of gross motor skills. In China, where the physical education market is rapidly expanding (52), optimizing early childhood education scientifically can not only enhance the quality of education but also create new job opportunities.

Although the benefits of targeted motor skill exercise training for preschool children have been well-documented, we must also acknowledge the practical challenges involved. It is important to note that the teacher-directed programs in the included studies were either supervised by researchers or achieved after early childhood education teachers underwent standardized training courses. At the practical level, teachers working in early childhood education are generalists: They are trained in various aspects of early childhood education but are not specialized in PE. Therefore, the lack of specialized PE training presents a major barrier to the widespread adoption of intervention programs. To address this challenge, there are two potential approaches. The first approach, through policy intervention, involves requiring early childhood education teachers to obtain specialized PE certification. This model necessitates comprehensive policy reform, which can be challenging to implement initially but can lead to the widespread adoption of advanced pedagogical approaches later on. The second approach involves the commercialization of scientifically validated lessons, with preschools conducting their teacher training. This model is better suited for private schools with higher educational standards and financial resources, and it can yield immediate outcomes.

The studies included in the analysis underscored the significant role of teacher-led targeted exercise training. Additionally, it is crucial to acknowledge that the majority of motor skill training requires specialized equipment (15, 20, 31, 35, 37, 39, 40, 43, 45), necessitating policy-level support. In China, physical activities in kindergartens mainly involve free outdoor play that requires minimal or no specialized sports equipment. Consequently, these activities do not effectively facilitate adequate motor skill development. This barrier related to the physical environment may be even noticeable in underdeveloped regions (53, 54). Therefore, policymakers need to coordinate financial allocations, such as one-time grants, to assist public kindergartens in acquiring appropriate sports equipment to enhance preschool children’s gross motor skills.

To ensure accurate and timely responses to developmental delays, it is advisable to establish a scientific standard for preschool-specific physical development. We recommend that Chinese policymakers conduct a nationwide assessment of gross motor skills, leading to the creation of a gross motor development curve for 3- to 6-year-old children in China, similar to Children’s general growth standards (55). Kindergarten teachers are advised to incorporate gross motor skills as a regular assessment item for children. Implementing a continuous monitoring system, such as a Chinese child gross motor development curve, will enable the identification of preschool children lagging behind in motor skills development and allow for tailored skill-enhancing training. This approach could also assist teachers in adjusting training courses (e.g., difficulty levels, exercise intensity) to match children’s capacities throughout an academic year.

In conclusion, existing studies offer compelling evidence regarding the effectiveness of motor development-focused exercise training compared to both free play and PE curriculum. Therefore, preschools are advised to organize systematic and teacher-guided complex physical activities to improve the gross motor skills of children aged 3 to 6 years.

## Data availability statement

The datasets presented in this study can be found in online repositories. The names of the repository/repositories and accession number(s) can be found at: https://doi.org/10.6084/m9.figshare.25545703.v1.

## Author contributions

XW: Conceptualization, Data curation, Formal analysis, Validation, Writing – original draft. BZ: Conceptualization, Data curation, Funding acquisition, Methodology, Project administration, Supervision, Writing – review & editing.
